# HIV-1 pretreatment drug resistance and genetic transmission network in the southwest border region of China

**DOI:** 10.1186/s12879-022-07734-3

**Published:** 2022-09-19

**Authors:** Difei Li, Huichao Chen, Huilan Li, Yanling Ma, Lijuan Dong, Jie Dai, Xiaomei Jin, Min Yang, Zhijun Zeng, Pengyan Sun, Zhizhong Song, Min Chen

**Affiliations:** 1 School of Public Health, Kunming Medical University, Kunming, Yunnan China; 2 Institute for AIDS/STD Control and Prevention, Yunnan Center for Disease Control and Prevention, No 158, Dongsi Street, Xishan District, Kunming, 650022 Yunnan Province China; 3 Division for AIDS/STD Control and Prevention, Pu’er Center for Disease Control and Prevention, Pu’er, Yunnan China

**Keywords:** HIV, Pretreatment drug resistance, Genetic transmission network

## Abstract

**Background:**

HIV drug resistance increased with the widespread use of antiretroviral drugs, and posed great threat to antiretroviral therapy (ART). Pu’er Prefecture, lying in the southwest of Yunnan Province, China, borders Myanmar, Laos and Vietnam, is also the area where AIDS was discovered earlier, however, in which there has been no information on HIV drug resistance.

**Methods:**

A cross-sectional survey of pretreatment drug resistance (PDR) was conducted in Pu’er Prefecture in 2021. Partial *pol* gene sequences were obtained to analyze drug resistance and construct genetic transmission network. HIV drug resistance was analyzed using the Stanford University HIVdb algorithm.

**Results:**

A total of 295 sequences were obtained, among which 11 HIV-1 strain types were detected and CRF08_BC (62.0%, 183/295) was the predominant one. Drug resistance mutations (DRMs) were detected in 42.4% (125/295) of the sequences. The prevalence of PDR to any antiretroviral drugs, nucleoside reverse transcriptase inhibitors (NRTIs), non-nucleoside reverse transcriptase inhibitors (NNRTIs) and protease inhibitors (PIs) were 10.8% (32/295), 9.5% (28/295), 1.0% (3/295) and 0.3% (1/295), respectively. The risk of PDR occurrence was higher among individuals with CRF01_AE strain types. HIV-1 molecular network was constructed, in which 56.0% (42/75) of links were transregional, and 54.7% (41/75) of links were associated with Lancang County. Among the sequences in the network, 36.8% (35/95) harbored DRMs, and 9.5% (9/95) were drug resistance strains. Furthermore, 8 clusters had shared DRM.

**Conclusion:**

The overall prevalence of PDR in this study was in a moderate level, but NNRTIs resistance was very approaching to the threshold of public response initiation. PDR was identified in the transmission network, and DRMs transmission was observed. These findings suggested that the consecutive PDR surveillance should be conducted in this region.

**Supplementary Information:**

The online version contains supplementary material available at 10.1186/s12879-022-07734-3.

## Background

Since the widespread application of highly active antiretroviral therapy (HAART), HIV incidence and HIV-associated morbidity and mortality has fallen considerably [1, 2]. However, the emergence of HIV drug resistance (HIVDR) has compromised the efficacy of antiretroviral therapy (ART) [3]. A series of studies have demonstrated that individuals with a consistent undetectable HIV viral load for at least 6 months cannot transmit HIV to others, which is called undetectable equals untransmittable (U = U) [4–6]. The increased ART failure caused by HIVDR not only impairs the health of people living with HIV/AIDS (PLWHA), but also results in the spread of resistant strains, which poses a great challenge to the controlling of HIV epidemic. To respond to the threat of emerging HIVDR, it is necessary to monitor the prevalence of drug resistance in the population initiating ART, which provides information for the selection of first-line ART regimens.

According to HIV drug resistance report published by World Health Organization (WHO) in 2021, there were 27.5 million people receiving ART globally, 21 of 30 reported surveys showed that the prevalence of pretreatment HIV drug resistance (PDR) to nevirapine (NVP) or efavirenz (EFV) in populations initiating first-line ART exceeded 10%, ranged from 10.9% in Zimbabwe to 27% in EI Salvador [7]. Mathematical modeling predicts that, if the prevalence of pretreatment HIVDR exceeds 10% in sub-Saharan Africa and non-nucleoside reverse transcriptase inhibitors (NNRTIs) continue to be used in the present first-line ART regimens, in the next 15 years, pretreatment HIVDR is predicted to be responsible for cumulatively 890,000 AIDS-related deaths and 150,000 new HIV infections in sub-Saharan Africa only [8]. As individual-level HIVDR testing is unaccessible in many low- and middle-income countries, nationally or regionally representative pretreatment HIVDR data can be used to inform when public health actions should be taken at population level, such as the switch of first-line ART regimens [9]. According to the WHO guideline, when the prevalence of pretreatment NNRTI resistance reaches 10% in NNRTI-containing first-line ART countries or regions, a non-NNRTI-containing regimen is recommended [10].

Currently, the main regimens of first-line ART in China consist of two NRTIs and one NNRTI. When the first-line ART fails, at least two drugs will be switched into susceptible ones [11]. Even susceptible protease inhibitors (PIs) or integrase inhibitors (INSTIs) are recommended, NNRTIs can also be included in the switched regimens. A systematic review and meta-regression analysis found that the prevalence of HIVDR to any drugs and NNRTIs in ART-naïve population were in an increasing trend, from 3.75 and 1.75% in 2012 to 6.25% and 5.0% in 2017 respectively [12]. A nationwide survey conducted in 2017 revealed that the prevalence of PDR was 6.8% in China, and PDR to NNRTIs was 4.9% [13]. The recent researches showed that the prevalence of PDR in some areas of China reached 10%, such as 12.2% in Liangshan prefecture of Sichuan Province in 2017, 11.5% in Tianjin during 2016–2019, and 10.54% in Chongqing during 2018–2021 [13–15].

Yunnan is one of the regions burden the most severe HIV epidemic, which located at the southwest of China with eight prefectures border Southeast Asian countries, including Myanmar, Laos and Vietnam. Among these prefectures, Pu’er is the only one bordering all the three countries, which included 10 counties. HIV epidemic was first reported in Pu’er in 1991. By the end of 2020, the number of PLWHA in Pu’er Prefecture was 6415. A study found that the prevalence of HIVDR in ART-naïve HIV-1 infected entering travelers from Myanmar to Yunnan was up to 12.8% [16]. Previous studies showed that the prevalence of PDR in some areas of Yunnan that border Myanmar were approaching 10%, like 7.5% in Lincang city and 9.3% in Dehong Prefecture [13, 17]. Even Pu’er located at such a crucial position, the HIVDR status is still unknown yet. To understand the prevalence of PDR in Pu’er, a cross-sectional study was conducted there.

## Methods

### Study design and study participants

In 2021, a total of 390 PLWHA initiated and reinitiated ART in Pu’er Prefecture of Yunnan Province, among which 339 were recruited. Eligibility criteria were as follows: initiated or reinitiated ART (interrupting ART at least 3 months) in 2021; Aged ≥ 15 years; and provided signed informed consent. The relative information was collected, including sex, age, ethnicity, occupation, marital status, registered address, history of ART exposure, infected route and baseline CD4^+^ T lymphocyte count. Blood samples were collected before initiating ART. The study was approved by the Biomedical Ethics Review Committee of Yunnan Center for Disease Control and Prevention (YNCDC-EA202107). All methods were carried out in accordance with relevant guidelines and regulations. The informed consent was obtained from all subjects and/or their legal guardian (s).

### Extraction and amplification of HIV-1 *pol* genes

Blood plasma samples were preserved at − 80 °C before RNA extraction. Viral RNA was extracted from plasma using QIAmp Viral RNA Mini Kit (Qiagen Germany) according to the manufacturer protocol. Partial *pol* sequence that corresponded to codons 1 to 99 of protease and codons 1–299 of reverse transcriptase was amplified by in-house nested reverse transcription PCR. The cDNA synthesis and first-round PCR operation were performed with One Step RNA PCR Kit (Takara, China). Cycling conditions were 50 °C for 30 min; 94 °C for 5 min; 94 °C for 30 s, 55 °C for 30 s, 72 °C for 2.5 min, 30 cycles; followed with an extension at 72 °C for 10 min. The nested PCR was performed with 2 × PCR Mix (TIANGEN, China). Cycling conditions were 94 °C for 5 min; 94 °C for 30 s, 63 °C for 30 s, 72 °C 2.5 min, 30 cycles; followed with an extension at 72 °C for 10 min. PCR products were visualized by 1% agarose gel electrophoresis. Successfully amplified samples were sequenced by Biomed Co. (Beijing, China) on an ABI 3730xl automated DNA analyzer (Applied Biosystems, Foster City, CA). The primers for PCR and sequencing are listed in Additional file 1: Table S1.

### Identification of HIV-1 genotypes and analysis of drug resistance

The sequences were assembled using Sequencher 5.1 (Gene Codes, Ann Arb-or, MI). Bio-Edit 7.0 software was used for ClustalW multiple alignment and manual editing. HIV-1 genotypes were identified by the REGA HIV-1 Subtyping Tool-Version 3.0 (http://dbpartners.stanford.edu:8080/RegaSubtyping/stanford-hiv/typingtool/) and COntext-based Modeling for Expeditious Typing tool (COMET HIV-1) (http://comet.retrovirology.lu). Drug resistance mutations (DRMs) and resistance levels were determined based on the Stanford HIVdb Program (https://hivdb.stanford.edu/hivdb/by-sequences/). According to the WHO judgment criteria for PDR, a sequence sample that has low, intermediate or high level resistance to the listed 12 drugs is defined as drug resistant, including two NNRTIs (EFV and NVP), seven nucleotide reverse transcriptase inhibitors (NRTIs, abacavir (ABC), zidovudine (AZT), stavudine (D4T), didanosine (DDI), emtricitabine (FTC), lamivudine (3TC), tenofovir (TDF)) and three proteinase inhibitors (PIs, atazanavir/r (ATV/r), darunavir/r (DRV/r) and lopinavir/r (LPV/r)) [18].

### Analysis of HIV-1 molecular network

A locally installed HIV TRAnsmission Cluster Engine (HIV-TRACE) was used to identify HIV-1 molecular network, which is based on a pairwise genetic distance threshold under Tamura-Nei 93 (TN93) nucleotide substitution model [19]. The pilot analysis was performed under the pairwise genetic distances varying from 0.1% to 2.0% with an interval of 0.1%. As shown in Additional file 1: Figure S1, the number of clusters reached the maximum at the genetic distance of 1.3%. Thus, a genetic distance threshold of 1.3% was used in this study. The sequences with a genetic distance of ≤ 1.3% were considered to be potential transmission links.

### Statistical analysis

Statistical analysis was conducted using the SPSS 19.0 software (SPSS Inc. Chicago, IL). All collected information were appropriately transformed into categorical variables, and was described by numbers and percentages. Chi-square test was used to compare the characteristics of different HIV strain types. Univariate and multivariate logistic regression models were used to analyze the potential factors that associated with PDR. All tests were two tailed, and *p* value < 0.05 was considered significant.

## Results

### General characteristics of the participants

A total of 339 participants were enrolled before initiating or reinitiating ART in Pu’er Prefecture in 2021. Of them, 295 partial *pol* gene sequences were successfully amplified. The proportion of successful amplification was 87.0% (295/339). The demographic characteristics showed no significant differences between the 295 individuals and the total 339 participants (Additional file 1: Table S2).

Of the 295 individuals with *pol* sequences, the average age was 39.7 ± 12.0 years old (range from 15 to 74); 68.5% (202/295) were male and 31.5% (93/295) were female; 78.3% (231/295) were farmers and 21.7% (64/295) were other occupation; 8.8% (26/295) had ART exposed history and 91.2% (269/295)were ART naive; for the ethnicity, Han accounted for 39.3% (116/295), the others accounted for 60.7% (179/295), including Lahu, Wa, Hani, Dai, Yi, Hui, Yao, Bulang and Menggu; for education levels, illiteracy, primary school, junior high school and senior high school or above accounted for 12.5% (37/295), 41.7% (123/295), 33.9% (100/295) and 11.9% (35/295) respectively; for marital status, unmarried, married and divorced or widowed accounted for 44.4% (131/295), 36.9% (109/295) and 18.7% (55/295) respectively; for HIV transmission routes, heterosexual contact, homosexual contact and intravenous drug use accounted for 83.7% (247/295), 7.1% (21/295) and 9.2% (27/295) respectively. The CD4^+^T lymphocyte counts were divided into four levels (< 200, 200–349, 350–499 and ≥ 500), accounted for 29.2% (86/295), 28.8% (85/295), 20.0% (59/295) and 14.6% (43/295) respectively, and 7.6% (22/295) missed the CD4^+^ T lymphocyte counts.

### The distribution of HIV-1 genotypes

Among the 295 successfully amplified sequences, 11 HIV-1 strain types were detected. CRF08_BC was the predominant genotype, accounting for 62.0% (183/295), followed by CRF01_AE (17.6%, 52/295), CRF07_BC (15.6%,46/295), URF strains (1.4%, 4/295), CRF55_01B (1.0%, 3/295), subtype C (0.7%, 2/295), subtype B (0.3%, 1/295), CRF118_BC (0.3%, 1/295), CRF59_01B (0.3%, 1/295), CRF68_01B (0.3%, 1/295) and CRF86_BC (0.3%, 1/295). URF strains included BC (75.0%, 3/4) and CRF01_AE/B (25.0%, 1/4).

The distribution of HIV-1 genotypes showed significant differences by registered residence, education level, occupation and HIV infection route (Table 1). Among the individuals from other provinces, CRF01_AE (42.9%, 9/21) was the predominant strain, while CRF08_BC was the predominant strain among the individuals from Pu’er Prefecture (64.8%, 164/253), other cities in Yunnan (58.8%, 10/17) and other countries (75.0%, 3/4). The proportion of CRF07_BC among the individuals with senior high school education or above was higher than that among those with junior high school education or below. The proportion of CRF08_BC among the farmers (68.8%, 159/231) significantly higher than that in the other sub-populations (37.5%, 24/64; χ^2^ = 20.888, p < 0.001). CRF08_BC was the predominant strain in the transmission routes of heterosexual contact (63.6%, 157/247) and intravenous drug use (88.9%, 24/27), while CRF07_BC was the predominant strain in homosexual contact (52.4%, 11/21).

**Table 1:** Demographic characteristics and HIV-1 genotypes of the study participants

Characteristics	Total	HIV-1 genotype				χ^2^	p
		CRF01_AE (%)	CRF07_BC (%)	CRF08_BC (%)	Others (%)		
Total	295 (100.0)	52 (17.6)	46 (15.6)	183 (62.0)	14 (4.7)		
Sex						7.540	0.061
Male	202 (100.0)	31 (15.3)	38 (18.8)	122 (60.4)	11 (5.4)		
Female	93 (100.0)	21 (22.6)	8 (8.6)	61 (65.6)	3 (3.2)		
Age						8.320	0.535
15–29	60 (100.0)	13 (21.7)	13 (21.7)	30 (50.0)	4 (6.7)		
30–39	99 (100.0)	13 (13.1)	14 (14.1)	66 (66.7)	6 (6.1)		
40–49	77 (100.0)	13 (16.9)	10 (13.0)	52 (67.5)	2 (2.6)		
≥ 50	59 (100.0)	13 (22)	9 (15.3)	35 (59.3)	2 (3.4)		
Ethnicity						2.633	0.454
Han	116 (100.0)	25 (21.6)	19 (16.4)	66 (56.9)	6 (5.2)		
Others	179 (100.0)	27 (15.1)	27 (15.1)	117 (65.4)	8 (4.5)		
Registered residence						19.674	0.007
P u’er	253 (100.0)	40 (15.8)	40 (15.8)	164 (64.8)	9 (3.6)		
Other cities in Yunnan	17 (100.0)	3 (17.6)	3 (17.6)	10 (58.8)	1 (5.9)		
Other provinces	21 100.0)	9 (42.9)	3 (14.3)	6 (28.6)	3 (14.3)		
Foreign nationality	4 (100.0)	0	0	3 (75.0)	1 (25.0)		
Marital status						5.126	0.548
Unmarried	131 (100.0)	21 (16.0)	24 (18.3)	80 (61.1)	6 (4.6)		
Married	109 (100.0)	24 (22.0)	16 (14.7)	65 (59.6)	4 (3.7)		
Divorced/widowed	55 (100.0)	7 (12.7)	6 (10.9)	38 (69.1)	4 (7.3)		
Education						17.817	0.037
Illiteracy	37 (100.0)	7 ( 18.9)	3 (8.1)	25 (67.6)	2 (5.4)		
Primary school	123 (100.0)	21 (17.1)	14 (11.4)	81 (65.9)	7 (5.7)		
Junior middle school	100 (100.0)	19 (19.0)	16 (16.0)	63 (63.0)	2 (2.0)		
Senior middle school and above	35 (100.0)	5 (14.3)	13 (37.1)	14 (40.0)	3 (8.6)		
Occupation						22.901	< 0.001
Farmers	231 (100.0)	37 (16.0)	28 (12.1)	159 (68.8)	7 (3.0)		
Others	64 (100.0)	15 (23.4)	18 (28.1)	24 (37.5)	7 (10.9)		
Infection route						41.053	< 0.001
Heterosexual contact	247 (100.0)	46 (18.6)	33 (13.4)	157 (63.6)	11 (4.5)		
Homosexual contact	21 (100.0)	5 (23.8)	11 (52.4)	2 (9.5)	3 (14.3)		
Intravenous drug use	27 (100.0)	1 (3.7)	2 (7.4)	24 (88.9)	0		

### The distribution of drug resistance mutations

Drug resistance mutations were detected in 42.4% (125/295) of the successfully amplified sequences. In detail, 39.3% (116/295) harbored NNRTI resistance-associated mutation, 1.7% (5/295) harbored NRTI resistance-associated mutations and 4.4% (13/295) harbored PI resistance-associated mutations; 1.0% (3/295) harbored NNRTI and NRTI resistance-associated mutations, 1.4% (4/295) harbored NNRTI and PI resistance-associated mutations, and 0.3% (1/295) harbored NNRTIs, NRTIs and PI resistance-associated mutations.

As shown in Table 2, among all the detected DRMs, the most common mutations included V179D (13.9%, 41/295), E138A (5.8%, 17/295), V106I (4.7%, 14/295), K103N (4.7%, 14/295), V179E (4.7%, 14/295) and V179T (3.7%, 11/295). Except V106I, the distribution of the other five mutations showed significant differences among the different genotypes, V179D and E138A were mostly found in CRF08_BC and other genotypes, K103N was mostly found in CRF01_AE and CRF07_BC, V179E was mostly found in other genotypes, V179T was mostly found in CRF01_AE.

**Table 2:** The drug resistance mutations detected among the participants

DRM	CRF01_AE (%)	CRF07_BC (%)	CRF08_BC (%)	others (%)	Total (%)
Total	52	46	183	14	295
NNRTI					
V179D	0	2 (4.3)	37 (20.2)	2 (14.3)	41 (13.9)
E138A	0	0	16 (8.7)	1 (7.1)	17 (5.8)
V106I	4 (7.7)	1 (2.2)	8 (4.4)	1 (7.1)	14 (4.7)
K103N	6 (11.5)	3 (6.5)	5 (2.7)	0	14 (4.7)
V179E	2 (3.8)	2 (4.3)	8 (4.4)	2 (14.3)	14 (4.7)
V179T	8 (15.4)	0	3 (1.6)	0	11 (3.7)
P225H	2 (3.8)	1 (2.2)	0	0	3 (1.0)
E138G	1 (1.9)	0	1 (0.5)	1 (7.1)	3 (1.0)
A98G	0	0	2 (1.1)	0	2 (0.7)
V179DE	0	1 (2.2)	1 (0.5)	0	2 (0.7)
E138Q	0	0	1 (0.5)	0	1 (0.3)
G190A	1 (1.9)	0	0	0	1 (0.3)
K101E	1 (1.9)	0	0	0	1 (0.3)
V106M	0	1 (2.2)	0	0	1 (0.3)
V179EK	0	0	0	1 (7.1)	1 (0.3)
V179IT	1 (1.9)	0	0	0	1 (0.3)
V179DE	0	0	1 (0.5)	0	1 (0.3)
V179DIN	0	0	1 (0.5)	0	1 (0.3)
V108I	1 (1.9)	0	0	0	1 (0.3)
Y181C	1 (1.9)	0	0	0	1 (0.3)
NRTI					
A62V	0	0	1 (0.5)	0	1 (0.3)
M41L	0	0	1 (0.5)	0	1 (0.3)
T215I	0	1 (2.2)	0	0	1 (0.3)
T69G	1 (1.9)	0	0	0	1 (0.3)
M184I	0	0	1 (0.5)	0	1 (0.3)
PI accessory					
I47R	1 (1.9)	0	0	0	1 (0.3)
I47T	1 (1.9)	0	0	0	1 (0.3)
I47K	1 (1.9)	0	0	0	1 (0.3)
K43T	0	1 (2.2)	0	0	1 (0.3)
L10F	1 (1.9)	0	0	0	1 (0.3)
L33F	2 (3.8)	0	0	0	2 (0.7)
M46T	0	0	1 (0.5)	0	1 (0.3)
Q58E	0	1 (2.2)	1 (0.5)	0	2 (0.7)
G48R	0	0	1 (0.5)	0	1 (0.3)
G73S	0	0	1 (0.5)	0	1 (0.3)
PI					
I50V	0	0	1 (0.5)	0	1 (0.3)

### Prevalence of PDR and associated factors

According to WHO discrimination criteria for PDR, 32 samples were HIV-1 drug resistant strains, the prevalence of PDR was 10.8% (32/295, 95% *CI* 7.3–14.3%). The prevalence of PDR to NNRTIs, NRTIs and PIs were 9.5% (28/295, 95% *CI* 6.2–12.8%), 1.0% (3/295, 95% *CI* 0–2.1%), and 0.3% (1/295, 95% *CI* 0–0.9%), respectively. Multiple resistance to two or three categories of antiretrovial drugs were not found in this study. The prevalence of PDR in ART naïve individuals (10.0%, 27/269) and that in ART experienced ones (19.2%, 5/26) showed no significant difference (χ^2^ = 1.23, p = 0.267).

The factors associated with PDR were further analyzed (Table 3). The multivariate logistic regression analysis showed PDR was most likely found in individuals with CRF01_AE (OR 3.14, 95% CI 1.29–7.63).

**Table 3:** The factors associated with drug resistance among the participants

Variable	Number	PDR N (%)	Univariate analysis		Multivariate analysis	
			OR (95%CI)	p	aOR (95%CI)	p
Total	295	32 (10.8)				
Sex						
Male	202	20 (9.9)	1.00	–		
Female	93	12 (12.9)	1.35 (0.63–2.89)	0.442		
Age				0.489		
15–29	60	5 (8.3)	1.00	–		
30–39	99	12 (12.1)	1.52 (0.51–4.54)	0.456		
40–49	77	6 (7.8)	0.93 (0.27–3.21)	0.908		
≥ 50	59	9 (15.3)	1.98 (0.62–6.31)	0.248		
Ethnicity						
Others	179	17 (9.5)	1.00	–		
Han	116	15 (12.9)	1.42 (0.68–2.96)	0.356		
Registered residence				0.775		
Pu’er	253	26 (10.3)	1.00	–		
Other cities in Yunnan	17	3 (17.6)	1.87 (0.50–6.94)	0.349		
Other provinces	21	3 (14.3)	1.46 (0.40–5.28)	0.568		
Foreign countries	4	0	0.00	0.999		
Marital status				0.486		
Unmarried	131	11 (8.4)	1.00	–		
Married	109	14 (12.8)	1.61 (0.70–3.70)	0.265		
Divorced/widowed	55	7 (12.7)	1.59 (0.58–4.35)	0.365		
Education				0.931		
Illiteracy	37	3 (8.1)	1.00	–		
Primary school	123	13 (10.6)	1.34 (0.36–4.98)	0.663		
Junior middle school	100	12 (12.0)	1.55 (0.41–5.82)	0.52		
Senior middle school and above	35	4 (11.4)	1.46 (0.30–7.06)	0.636		
Occupation						
Farmers	231	21 (9.1)	1.00	–	1.00	–
Others	64	11 (17.2)	2.08 (0.94–4.57)	0.070	1.86 (0.80–4.31)	0.149
Infection route				0.772		
Injected drug use	27	2 (7.4)	1.00	–		
Heterosexual contact	247	30 (12.1)	1.73 (0.39–7.67)	0.472		
Homosexual contact	21	0	0	0.998		
Strain type				0.057		0.074
CRF08_BC	183	14 (7.7)	1.00	–	1.00	–
CRF07_BC	46	6 (13.0)	1.81 (0.66–5.00)	0.252	1.61 (0.56–4.63)	0.382
CRF01_AE	52	11 (21.2)	3.24 (1.37–7.66)	0.007	3.14 (1.29–7.62)	0.012
Others	14	1 (7.1)	0.93 (0.11–7.63)	0.945	0.74 (0.09–6.40)	0.781
ART exposure						
Naive	269	27 (10.0)	1.00	–	1.00	–
Exposed	26	5 (19.2)	2.13 (0.74–6.12)	0.158	2.53 (0.85–7.54)	0.095
Baseline CD4 (cells/µl)				0.486		
< 350	171	17 (9.9)	1.00	–		
≥ 350	102	14 (13.7)	1.61 (0.70–3.70)	0.265		
Unknown	22	1 (4.5)	1.59 (0.58–4.35)	0.365		

### Drug resistance in HIV-1 molecular network

**Fig. 1:**
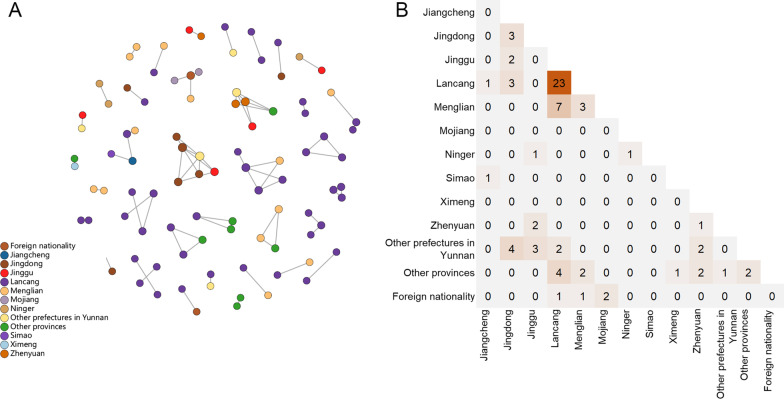
The interregional HIV-1 genetic transmission networks. **A** HIV-1 molecular network coded by the individuals’ registered residence. **B** The matrix of HIV-1 genetic links between the different regions. The cells on the diagonal represented the number of the links in the same region. The other cells represented the number of the links between the different regions

As shown in Fig. 1, 95 (32.2%) sequences were included into 36 clusters with a genetic distance threshold of 0.013, which identified the maximum number of clusters. Among these clusters, the number of nodes ranged from 2 to 6. Of the 75 links in the network, 44.0% (33/75) occurred between the individuals with the same registered residence (Fig. 1A), which were on the diagonal in Fig. 1B, while 56.0% (42/75) made up of the individuals from different registered residence, which were not on the diagonal in Fig. 1B. It was worthy of attention that 54.7% (41/75) links were associated with Lancang County.

**Fig. 2:**
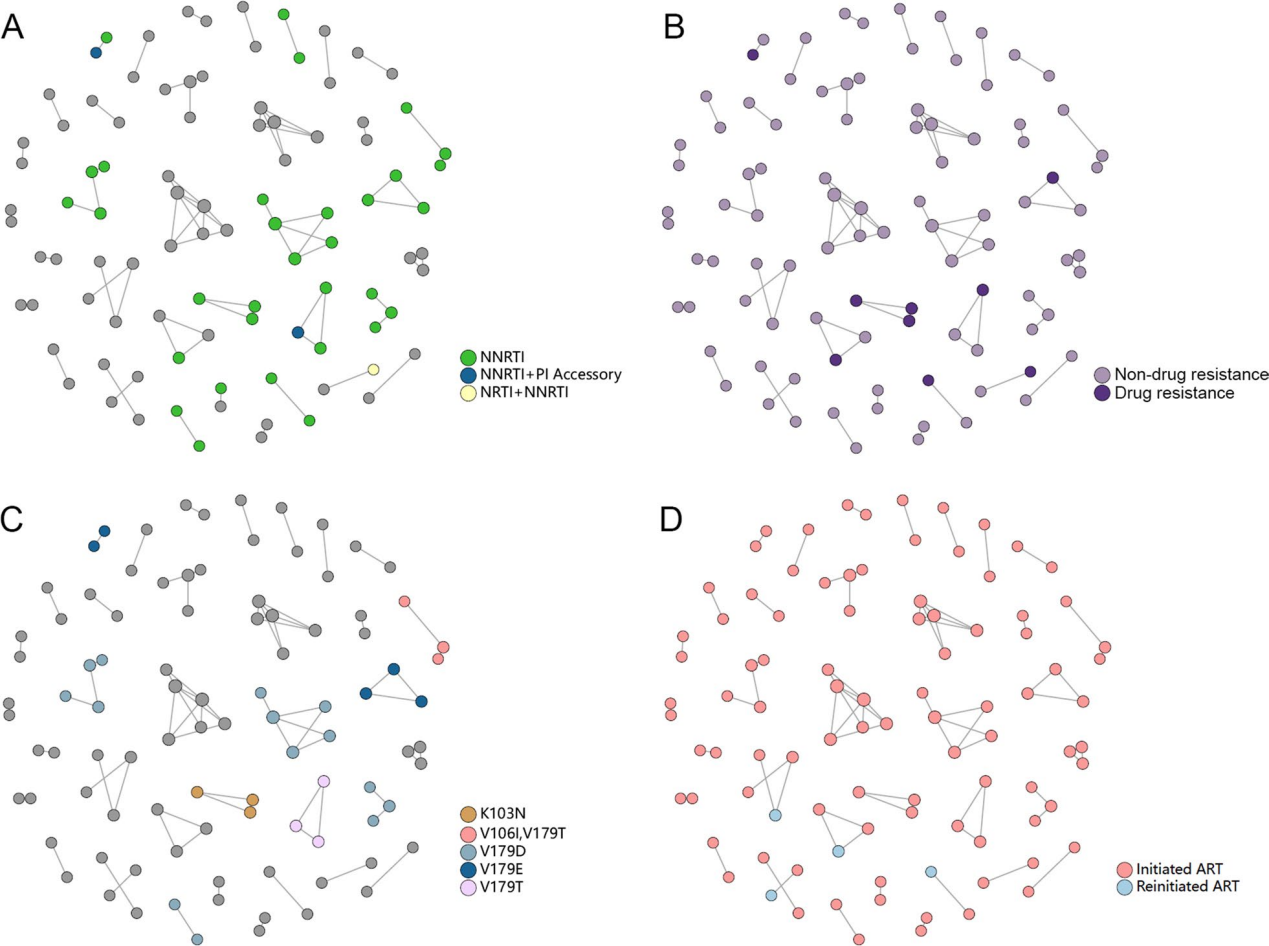
The drug resistance in HIV-1 molecular network. **A** The DRM types of the nodes in HIV-1 molecular network. **B** The nodes with drug resistance in HIV-1 molecular network. **C** The nodes with shared DRM in HIV-1 molecular network. **D** The nodes representing PLWHA who initiated and reinitiated ART

In the molecular network, 36.8% (35/95) sequences harbored DRMs (Fig. 2A), and 9.5% (9/95) were drug resistant strains (Fig. 2B). In addition, 8 clusters had shared DRM (Fig. 2C), including K103N in one cluster, V106I/V179T in one cluster, V179D in three cluster, V179E in two cluster and V179T in one cluster. Further, four individuals reinitiating ART were found in four different clusters (Fig. 2D). Of the four sequences, two were drug resistant strains (Fig. 2B and D). However, these four clusters had no shared DRM (Fig. 2C and D).

## Discussion

In this study, a cross-sectional pre-treatment drug resistance survey was performed in Pu’er Prefecture, one of the border prefectures in Yunnan Province, which revealed the distribution of HIV-1 genotypes and the prevalence of PDR among the individuals initiating or reinitiating ART in this area.

Located in the southwest of Yunnan Province, Pu’er Prefecture borders Myanmar in the west and Laos and Vietnam in the east, and is near the "Golden Triangle", the world’s largest drug production center. In history, the drug trafficking routes also passed through Pu’er Prefecture. The intravenous drug use promoted the development of the early HIV-1 epidemic in the local area, which further transited to the general population through heterosexual contact [20]. Thus, the border areas of Yunnan Province are all severely affected by HIV. Meanwhile, the China-Myanmar border area constitutes a hot spot of active recombination in Asia, more than a dozen of novel CRFs were discovered in this area in recent years, which suggested the HIV epidemic is complex.

A total of 11 HIV-1 genotypes were found in this study, which revealed HIV-1 genetic diversity in Pu’er. CRF08_BC, CRF01_AE and CRF07_BC were the dominant strains in Pu’er, which was similar to the circulating characteristics of HIV-1 genotypes in Yunnan Province [21]. Historically, CRF01_AE was first reported in female sex workers returning from Thailand to Pu’er [22], which was also the first confirmation of CRF01_AE in Yunnan. In the early stage, CRF01_AE was most confined to the western prefectures in Yunnan, including Pu’er [23], CRF07_BC and CRF08_BC were most found in the eastern region of Yunnan [23]. In the late 2000s, the prevalence of CRF08_BC and CRF07_BC increased in the western areas [24]. As found in this study, the proportion of CRF08_BC was far higher than the other HIV-1 strains. Besides the above three CRFs, some novel CRFs were found, which were first identified in other prefectures and other provinces. This suggested that the presence of transregional transmission.

The HIV distribution of HIV-1 genotypes showed difference by registered residences. CRF08_BC was the predominant strain among the Pu’er natives, individuals from others cities in Yunnan and foreigners. However, CRF01_AE was the predominant strain among individuals from other provinces. One possibility was that individuals from other provinces were infected before entering Yunnan, the other was that they were involved in the specific risk network after entering Yunnan. The different transmission routes had their own predominant HIV-1 strains, the proportion of CRF08_BC was higher in the individuals infected through heterosexual contact and intravenous drug use, that of CRF07_BC was higher in MSM, which was similar to the prevalence of HIV-1 genotypes in Yunnan [21]. The distribution of HIV-1 genotypes also showed significant differences by education level and occupation. The further analysis found the constitutions of transmission routes were different when stratified by education levels and occupation (Additional file 1: Tables S3 and S4). As observed, the proportion of CRF07_BC was significantly higher in individuals with senior middle school education or above and non-farmers, probably because the proportion of homosexual contact was significantly higher in these two sub-populations (Additional file 1: Tables S3 and S4). As mentioned above, CRF07_BC was the predominant strain in homosexual contact.

Among the 295 participants, 42.4% were found to carry DRMs, which was higher than the similar studies [14]. The proportion of participants carried NNRTI resistance-associated mutations was far higher compared with the proportion of participants carried NRTI and PI resistance-associated mutation, which was consistent with the similar studies [17]. HIV-1 has a low genetic barrier to NNRTIs resistance, namely a single mutation in the binding site may cause drug resistance. Thus, NNRTIs resistance-associated mutations most frequently occurred [23]. Among the DRM detected, the top six were NNRITs associated mutations, of which V179D, E138A, K103N, V179E and V179T showed HIV-1 genotype specific distribution, which suggested that the different strains had their own drug resistance characteristics. Most DRM detected alone would not cause resistance or only caused potential low level drug resistance, when combining with additional mutations, they act synergistically to reduce drug susceptibility. As found in this study, the combinations of E138G/V179D, E138G/V179E, V108I/V179T and V106I/V179D cause resistance to NNRTIs. Being detected at a high frequency, K103N is also a key DRM, which confers high level resistance to EFV and NVP independently. In addition, there were some DRMs being detected at a low frequency, but they could confer resistance directly, such as P225H, A98G, K101E, Y181C, M41L, T215I and I50V. These resistant strains also need attention.

The overall prevalence of PDR in Pu’er was 10.8%, which was in a moderate level according to WHO’s definition of about the HIV drug resistance level. A nationwide PDR survey showed that the prevalence of PDR in China was 6.8%, and other studies showed that the prevalence of PDR in Guangxi Province and Lincang prefecture of Yunnan province were 8.3% and 7.5% respectively [13, 17, 25]. The prevalence of PDR in Pu’er was higher when compared with them. The risk of PDR occurrence was higher among patients with CRF01_AE than those with other strain types. This may be related to the fact that CRF01_AE has been prevalent in this area for a long time. A series studies demonstrated that prevalence of PDR was higher among individuals with prior ART exposure than those without prior ART exposure [26]. Our study also found the higher prevalence of PDR among prior ART exposed individuals, but the difference was not significant in statistics. The prevalence of PDR to NNRTIs was 9.8%, which was approaching to the threshold of 10%. According to WHO guideline, when the prevalence of PDR to NNRTI reached 10% and above, in response, a non-NNRTI-containing regimen was recommended. In 2018, WHO published interim guidelines that recommended dolutegravir (DTG) as a preferred component of first-line treatment to replace EFV and NVP. According to WHO published “Global action plan on HIV drug resistance 2017–2021”, 26 countries which reported a PDR prevalence exceeding 10% had initiated the transition to DTG-based first-line ART. In addition, 10 African countries which reported high level resistance to EFV and NVP had adopted LPV/r as the preferred first-line regimen [27]. Pu’er Prefecture is the area where HIV/AIDS cases were reported earlier in Yunnan Province. The prolonged epidemics could increase the accumulation of resistant mutations. In order to cope with the further development of drug resistance epidemic, it is necessary to strengthen drug resistance surveillance and treatment effectiveness evaluation.

The potential associations between infected individuals were further analyzed using molecular network methods. More than half of links made up of the individuals from different registered residence, which suggested that these links were putative transregional transmission. Thus, in addition to preventive measures within the jurisdiction, coordinated prevention and control between regions also need to be considered. In particular, Lancang County was engaged into more than 50% of links, and became a regional hub of transmission. Due to the Coronavirus disease 2019 (COVID-19) pandemic, the number of newly reported HIV infected foreigners has decreased. However, four Burmese newly reported in 2021 all engaged into the molecular networks, which suggested that HIV/AIDS prevention and treatment for foreigners deserves attention. Furthermore, some clusters shared DRMs, which suggested that DRMs transmitted in the network. Although some shared DRMs did not directly cause resistance, three sequences harboring K103N in a cluster were drug resistance strains, which may be transmitted drug resistance. The individuals reinitiating ART were found in the four clusters, in which no shared DRMs were detected. Thus, the phenomenon that individuals interrupting ART caused drug resistance transmitting was not observed for the time being.

This study also has some limitations. First, not all participant’s HIV gene sequences were obtained, the failure of extraction and amplification might be due to viral RNA degradation caused by poor storage and transportation conditions of the samples. Second, the relatively short study period and small sample size limited the better construction of the transmission network.

## Conclusions

In summary, this study revealed the genetic diversity of HIV-1 and the prevalence of PDR in Pu’er. The overall prevalence of PDR was in a moderate level, but NNRTIs resistance was approaching the threshold of public response initiation. Shared DRMs and transmitted PDR were observed in the molecular clusters. To ensure the effectiveness of the first-line drug regimen, consecutive PDR surveillance should be further conducted in this area.

## Supplementary Information

**Additional file 1: Table S1.** Primers for PCR and sequencing in the study. **Fig. S1.** Evaluation of the effect of the genetic distance threshold on cluster identification. A, Number of clusters changing with an increasing of genetic distance threshold. B, Number of clustered sequences changing with an increase of genetic distance threshold. **Table S2.** The constituents of the subjects successfully genotyped. **Table S3.** The constitutions of transmission routes in the different education levels. **Table S4.** The constitutions of transmission routes in the different occupations.

## Data Availability

The sequences obtained in this study were submitted to NCBI GenBank under accession numbers ON736445-ON736739. The datasets are available from the corresponding author on reasonable request.

## Abbreviations

HAART: Highly active antiretroviral therapy
HIV: Human immunodefciency virus
ART: Antiretroviral therapy
HIVDR: HIV drug resistance
PLWHA: People living with HIV/AIDS
WHO: World Health Organization
PDR: Pretreatment drug resistance
DRM: Drug resistance mutation
NNRTI: Non-nucleoside reverse-transcriptase inhibitor
NRTI: Nucleoside reverse-transcriptase inhibitor
PI: Protease inhibitor
EFV: Efavirenz
NVP: Nevirapine
FTC: Emtricitabine
3TC: Lamivudine
ABC: Abacavir
AZT: Zidovudine
DDI: Didanosine
D4T: Stavudine
TDF: Tenofovir
ATV/r: Atazanavir/r
DRV/r: Darunavir/r
LPV/r: Lopinavir/r
DTG: Dolutegravir
OR: Odds ratio
aOR: Adjusted odds ratio
CI: Confidence interval
