# Supplementary material for: HIV-1 pretreatment drug resistance and genetic transmission network in the southwest border region of China
Source: BMC Infect Dis. 2022 Sep 19;22:741. doi: 10.1186/s12879-022-07734-3 (PMC9483295; doi:10.1186/s12879-022-07734-3)
Supplement: Supplementary file 1 — Additional file 1: Table S1. Primers for PCR and sequencing in the study. Fig. S1. Evaluation of the effect of the genetic distance threshold on cluster identification. A, Number of clusters changing with an increasing of genetic distance threshold. B, Number of clustered sequences changing with an increase of genetic distance threshold. Table S2. The constituents of the subjects successfully genotyped. Table S3. The constitutions of transmission routes in the different education levels. Table S4. The constitutions of transmission routes in the different occupations. [file 12879_2022_7734_MOESM1_ESM.docx]

**Additional file 1: Table S1. Primers for PCR and sequencing in the study**

| Procedure | Name (Direction) | Location (HXB2) | Sequences (5$'$-3$'$) |
| --- | --- | --- | --- |
| The first round RT-PCR | MAW 26 (F) | 2028 → 2050 | TTGGAAATGTGGAAAGGAAGGAC |
|  | RT21 (R) | 3539 → 3509 | CTGTATTTCTGCTATTAAGTCTTTTGATGGG |
| The second round PCR | PRO-1 (F) | 2147 → 2166 | CAGAGCCAACAGCCCCACCA |
|  | RT20 (R) | 3462 → 3441 | CTGCCAGTTCTAGCTCTGCTTC |
| Sequencing | PROS3 (F) | 2151 → 2166 | GCCAACAGCCCCACCA |
|  | PROC1S (R) | 3144 → 3128 | GCTGGGTGTGGTATTCC |
|  | RTB (F) | 2946 → 2967 | CCTAGTATAAACAATGAGACAC |
|  | RTAS (F) | 2524 → 2539 | CTCAGATTGGTTGCAC |
|  | RT20S3 (R) | 2524 → 2539 | CTCAGATTGGTTGCAC |


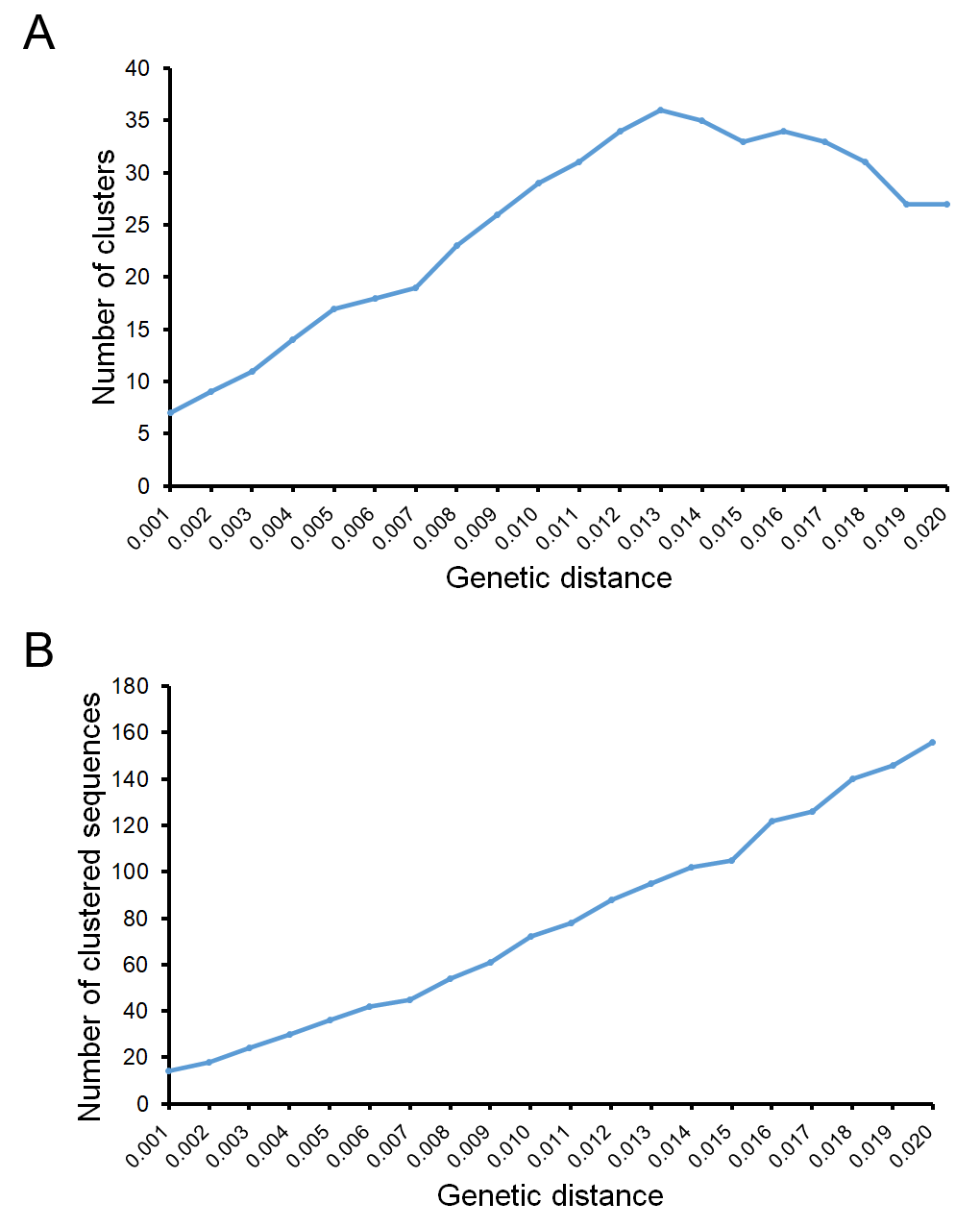


**Additional file 1: Figure S1.** Evaluation of the effect of the genetic distance threshold on cluster identification. A, Number of clusters changing with an increasing of genetic distance threshold. B, Number of clustered sequences changing with an increase of genetic distance threshold.

**Additional file 1: Table S2. The constituents of the subjects successfully genotyped.**

| Characteristics | | Subjects (%) | Subjects obtaining subtypes (%) | χ^2^ | P |
| --- | --- | --- | --- | --- | --- |
| Total | | 339 (100.0) | 295 (100.0) |  |  |
| Sex | |  |  | 0.324 | 0.606 |
|  | Male | 234 (69.0) | 202 (68.5) |  |  |
|  | Female | 105 (31.0) | 93 (31.5) |  |  |
| Age | |  |  |  | 0.778 |
|  | 15-29 | 69 (20.4) | 60 (20.3) |  |  |
|  | 30-39 | 116 (34.2) | 99 (33.6) |  |  |
|  | 40-49 | 89 (26.3) | 77 (26.1) |  |  |
|  | ≥50 | 65 (19.2) | 59 (20.0) |  |  |
| Ethnicity | |  |  |  | 0.535 |
|  | Han | 133 (39.2) | 116 (39.3) |  |  |
|  | Others | 206 (60.8) | 179 (60.7) |  |  |
| Registered residence | |  |  |  | 0.507 |
|  | Puer | 292 (86.1) | 253 (85.8) |  |  |
|  | Other cities in Yunnan | 20 (5.9) | 17 (5.8) |  |  |
|  | Other provinces | 22 (6.5) | 21 (7.1) |  |  |
|  | Foreign nationality | 5 (1.5) | 4 (1.4) |  |  |
| Marital status | |  |  |  | 0.977 |
|  | Unmarried | 150 (44.2) | 131 (44.4) |  |  |
|  | Married | 126 (37.2) | 109 (36.9) |  |  |
|  | Divorced/widowed | 63 (18.6) | 55 (18.6) |  |  |
| Education | |  |  |  | 0.427 |
|  | Illiteracy | 42 (12.4) | 37 (12.5) |  |  |
|  | Primary school | 141 (41.6) | 123 (41.7) |  |  |
|  | Junior middle school | 119 (35.1) | 100 (33.9) |  |  |
|  | Senior middle school and above | 37 (10.9) | 35 (11.9) |  |  |
| Occupation | |  |  |  | 0.434 |
|  | Farmers | 268 (79.1) | 231 (78.3) |  |  |
|  | Others | 71 (20.9) | 64 (21.7) |  |  |
| Infection route | |  |  |  | 0.81 |
|  | Heterosexual contact | 285 (84.1) | 247 (83.7) |  |  |
|  | Homosexual contact | 23 (6.8) | 21 (7.1) |  |  |
|  | Intravenous drug use | 31 (9.1) | 27 (9.2) |  |  |
| ART experience | |  |  |  | 0.403 |
|  | Naïve | 311 (91.7) | 269 (91.2) |  |  |
|  | Exposed | 28 (8.3) | 26 (8.8) |  |  |
| Baseline CD4 (cells/µl) | |  |  |  | 0.279 |
|  | ≥500 | 53 (15.6) | 43 (14.6) |  |  |
|  | 350-499 | 71 (20.9) | 59 (20.0) |  |  |
|  | 200-349 | 97 (28.6) | 85 (28.8) |  |  |
|  | <200 | 93 (27.4) | 86 (29.2) |  |  |
|  | Unknown | 25 (7.4) | 22 (7.5) |  |  |

**Additional file 1: Table S3. The constitutions of transmission routes in the different education levels.**

| education level | Total (%) | Heterosexual contact (%) | Homosexual contact (%) | Intravenous drug use (%) | χ^2^ | P |
| --- | --- | --- | --- | --- | --- | --- |
| Illiteracy | 37 (100.0) | 33 (89.2) | 1 (2.7) | 3 (8.1) | 28.106 | <0.001 |
| Primary school | 123 (100.0) | 110 (89.4) | 3 (2.4) | 10 (8.1) |  |  |
| Junior middle school | 100 (100.0) | 81 (81.0) | 6 (6.0) | 13 (13.0) |  |  |
| Senior middle school and above | 35 (100.0) | 23 (65.7) | 11 (31.4) | 1 (2.9) |  |  |

**Additional file 1: Table S4. The constitutions of transmission routes in the different occupations.**

| Occupation | Total | Heterosexual contact (%) | Homosexual contact (%) | Introvenous drug use (%) | χ^2^ | P |
| --- | --- | --- | --- | --- | --- | --- |
| Farmers | 231 (100.0) | 203 (87.9) | 5 (2.2) | 23 (10.0) | 31.409 | <0.001 |
| Others | 64 (100.0) | 44 (68.8) | 16 (25.0) | 4 (6.3) |  |  |
